# Passive Landmark Geometry Optimization and Evaluation for Reliable Autonomous Navigation in Mining Tunnels Using 2D Lidars

**DOI:** 10.3390/s22083038

**Published:** 2022-04-15

**Authors:** Miguel Torres-Torriti, Paola Nazate-Burgos, Fabián Paredes-Lizama, Javier Guevara, Fernando Auat Cheein

**Affiliations:** 1 Department of Electrical Engineering, Pontificia Universidad Católica de Chile, Santiago 782-0436, Chile; pjnazate@uc.cl (P.N.-B.); ftparede@uc.cl (F.P.-L.); 2 Department of Electronic Engineering, Universidad Técnica Federico Santa María, Valparaíso 239-0123, Chile; dario.guevara@sansano.usm.cl (J.G.); fernando.auat@usm.cl (F.A.C.)

**Keywords:** underground mining robots, scan matching, localization and SLAM in tunnels, 2D lidar navigation, GPS-denied environment

## Abstract

Autonomous navigation in mining tunnels is challenging due to the lack of satellite positioning signals and visible natural landmarks that could be exploited by ranging systems. Solutions requiring stable power feeds for locating beacons and transmitters are not accepted because of accidental damage risks and safety requirements. Hence, this work presents an autonomous navigation approach based on artificial passive landmarks, whose geometry has been optimized in order to ensure drift-free localization of mobile units typically equipped with lidar scanners. The main contribution of the approach lies in the design and optimization of the landmarks that, combined with scan matching techniques, provide a reliable pose estimation in modern smoothly bored mining tunnels. A genetic algorithm is employed to optimize the landmarks’ geometry and positioning, thus preventing that the localization problem becomes ill-posed. The proposed approach is validated both in simulation and throughout a series of experiments with an industrial skid-steer CAT 262C robotic excavator, showing the feasibility of the approach with inexpensive passive and low-maintenance landmarks. The results show that the optimized triangular and symmetrical landmarks improve the positioning accuracy by 87.5% per 100 m traveled compared to the accuracy without landmarks. The role of optimized artificial landmarks in the context of modern smoothly bored mining tunnels should not be understated. The results confirm that without the optimized landmarks, the localization error accumulates due to odometry drift and that, contrary to the general intuition or belief, natural tunnel features alone are not sufficient for unambiguous localization. Therefore, the proposed approach ensures grid-based SLAM techniques can be implemented to successfully navigate in smoothly bored mining tunnels.

## 1. Introduction

Improving underground mining productivity requires loaders with increased levels of autonomy in hauling and excavation tasks [1,2]. The capability of a machine to solve its pose both locally and globally in the network of tunnels is essential to achieve high autonomy levels.

The pioneering work [3] shows the feasibility of using simultaneous localization and mapping (SLAM) techniques to map old abandoned mines. At the heart of the approach in [3] is the scan matcher [4], which delivers locally consistent maps and estimates of the robot’s motion relying on the existence of structural elements, such as pillars and beams, that facilitate the data association. However, modern tunnel boring techniques produce smooth tunnels in hard rock or soft ground, with new techniques that even avoid traditional support pillars [5]. This makes correct scan matching infeasible without landmarks, unlike cases such as that of abandoned coal mines [3] or silver mines [6] several decades old. Hence, an approach is proposed here that employs optimized artificial *passive* landmarks that ensure correct data association for consistent mapping and can be cheaply manufactured, installed, require little or no maintenance, and may be easily replaced if damaged. Our results show that in environments such as smooth mining tunnels without landmarks, the localization problem becomes the ill-posed environments, and SLAM cannot be solved without the aid of landmarks or beacons, as noted by [7].

**Figure 1:**
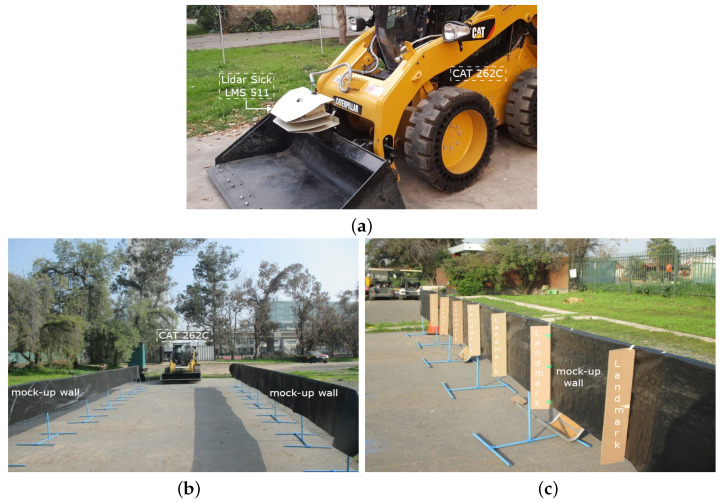
Experimental setup showing the semi-autonomous CAT 262C and the mock-up tunnel: (**a**) CAT 262C and the front lidar Sick LMS 511; (**b**) Tunnel mock-up without landmarks; (**c**) Tunnel mock-up with landmarks.

The design of a landmark’s optimized geometry and spacing is essential to ensure its identifiability, as well as an accurate and reliable localization of existing semi-autonomous tele-operated mining loaders equipped with standard 2D lidar scanners. Two landmark models are proposed and evaluated, one which employs shape primitives and another with a completely free shape described by a piecewise linear function. Hence, the main contributions of this work are in the landmark parameters’ optimization using a genetic algorithm, identifying suitable landmark shapes, and validating the localization approach for loaders in underground tunnels. The methodology was implemented in a simulated environment and validated experimentally using the semi-autonomous industrial CAT 262C skid-steer loader developed by the authors for research purposes [2]. The machinery used is shown in Figure 1a, and the mock-up tunnel used for experimental validation can be appreciated in Figure 1b,c.

The applicability of the landmark-based localization approach and SLAM in real mining environments is demonstrated in Section 5.3 using an accurate 3D model of a 100 m tunnel segment from the El Teniente mine located in Chile [8], which is the largest underground copper mine in the world.

This paper is organized as follows. Section 2 discusses the related work concerning autonomous navigation for robots in mining tunnels. Section 3 explains the preliminary mathematical notions of localization by scan matching. Section 4 presents the proposed approach, including the landmark models and their parametric description for optimization by means of a search strategy based on a genetic algorithm. The fifth section discusses the results obtained in simulations and experiments with an industrial skid-steer compact excavator CAT 262C. Finally, the discussion and conclusions are mentioned in Section 6 and Section 7, respectively.

## 2. Related Work

Since the first Automated Guided Vehicles (AGVs) were introduced to the market in 1950s, AGVs have evolved from pure wire or magnetic tape following mobile platforms into more sophisticated laser-guided vehicles (LGVs) or Autonomous Mobile Robots (AMRs). Navigation strategies have become particularly important because they enable the localization of mobile platforms by determining position and orientation. Early approaches used beacons, barcodes, and a combination of sensors for wheel odometry and evolving into more sophisticated laser scanning and vision systems for environmental recognition. Landmarks are useful for mobile robot navigation because they provide references for localization strategies that determine the position and orientation of mobile platforms. Humans use landmarks as a spatial representation of the environment to locate themselves and provide references to others. It is common to use landmarks to define the location of other objects/regions or in the creation of maps [9]. A landmark is a building, place, or object that is used for location and can be easily recognized. Robots, like humans, can recognize landmarks from their environment, and according to Thrun [10] landmarks can be those artificially placed in the environment or natural landmarks, which need to be discovered through algorithms to detect walls, corners, colors, etc. The first studies in the literature concerning localization methods for load-haul and dump (LHD) vehicles considered dead reckoning and artificial beacons [11]. The beacons used to correct the cumulative error from the odometry were retro-reflective markers in the roof detected using passive optical switches or passive LC (inductive-capacitive) resonators detected with simple antennas. Later developments included gyro sensors and laser-based guidance control systems (GCS). Gyros provide heading information, while GCS laser sensors allow locating the vehicles relative to the beacons using the beacons’ bearing angle [12]. This approach requires the beacons’ position to be known in the map the environment. An approach that combines RFID beacons as landmarks [13] combined with 2D lidar scanners for accurate mapping with the aim of using the maps in later localization technology.

Recent publications [14,15] propose the use of lidar-based SLAM approaches for autonomous navigation of LHDs. SLAM with laser scanners requires distinctive features in the environment that could serve as natural landmarks or anchor points without which the correct alignment of scans is not possible [16,17]. Unfortunately, unlike natural caves, mining tunnels are relatively straight or slowly curving, with walls that do not have distinctive uniquely identifiable elements, either because the walls have a repetitive coarse texture or are relatively smooth because modern tunnel boring machines and shotcrete spraying are employed in their construction.

Androulakis [18] uses a 2D lidar scanner to extract two types of features from pillar coal mine: linear segments for modeling entrance ribs and significant points for modeling intersection corners; however, between mine corners or entrances may be hundreds of meters where platforms will not have a robust reference or landmark. Therefore, landmarks, beacons, or some other kind of marker must be introduced in order to make SLAM-based approaches truly feasible. Based on the above, artificial landmarks can be justified for practical application in the navigation of mobile robots in harsh environments, and although the approach may be considered somewhat old and rudimentary, there are some basic issues in the design of optimized artificial landmarks that have not yet been resolved. In particular, the design of the optimized shape and spacing of landmarks must be studied to ensure their identifiability, as well as accurate and reliable localization of mobile robotic platforms. It is worth mentioning that the localization problem by means of scan matching can also be solved using different registration algorithms, such as the Iterative Closest Point (ICP) algorithm [19] or the Normal Distribution Transform (NDT) [20], but its analysis is out of the scope of this work. For a comparison of scan-matching approaches applied to the 3D mapping of underground mines, see, for example, the in-depth study by [21].

Others fields where landmarks are applied for robot localization include:

- Navigation in fruit groves with large tree canopies that create tunnel-like conditions [22];
- Visual place recognition (VPR) in changing environments for autonomous navigation exploiting landmarks to improve matching between images [23,24,25];
- Robotic guidance using sonar systems aided by landmarks that are inspired by the shape of flowers that act as sonar reflectors to improve localization and navigation [26];
- Indoor navigation [10,27];
- Outdoor urban navigation [28,29,30].

Methods using landmarks, such as poles or trees, for localization in GPS-denied agricultural and urban environments can be found in previous work by the authors [22,31] and references therein.

## 3. Preliminary Notions of Localization by Scan Matching

Let $q_{t}=\left(x_{t},y_{t},\theta_{t}\right)$ denote the pose vector of the mobile platform, composed by the position coordinates $\left(x_{t},y_{t}\right)$ in the 2D plane and its orientation $\theta_{t}$ measured with respect to the horizontal axis of the world reference frame at time *t*. Denote by $Z_{t}=\left\{\left(r_{t,k}^{s},\theta_{t,k}^{s}\right):k=1,2,\ldots ,m\right\}$ the set of lidar sensor measurements at time *t* expressed as a set of points with polar coordinates of the sensor’s frame of reference, which, for simplicity of exposition, is assumed to be coincident with the mobile platform’s frame of reference. Thus, $r_{t,k}^{s}$ represents the *k*-th measured distance from the mobile robot’s center of mass to the object reflecting the laser’s beam and $\theta_{t,k}^{s}$ the direction of the corresponding beam. For a platform that is estimated to be located at $\hat{q}=\left({\hat{x}}_{t},{\hat{y}}_{t},{\hat{\theta }}_{t}\right)$ at time *t*, the measurements in $Z_{t}$ imply that that the estimated locations of objects in the map have Cartesian coordinates in the world reference frame are given by:

$$\begin{array}{ccc} \hat{m}\left(\hat{q},Z_{t,k}\right) & = & \left[\begin{array}{c} \left(r_{t,k}^{s}+n_{r}\right)\cos\left({\hat{\theta }}_{t}+\theta_{t,k}^{s}+n_{\theta }\right)+{\hat{x}}_{t}+n_{x}\\ \left(r_{t,k}^{s}+n_{r}\right)\sin\left({\hat{\theta }}_{t}+\theta_{t,k}^{s}+n_{\theta }\right)+{\hat{y}}_{t}+n_{y} \end{array}\right] \end{array}$$

where $n_{r},n_{\theta }$ represent measurement noises in range and bearing, while $n_{x}$ and $n_{y}$ are position estimate uncertainties. Thus, the set of coordinates corresponding to the estimated location of objects in the world surrounding the robot is $M_{t}\left(\hat{q},Z_{t}\right)=\left\{\hat{z}\left(\hat{q},Z_{t,k}\right):k=1,2,\ldots ,m\right\}$. The robot localization problem can now be formulated as the problem of finding an estimated pose vector ${\hat{q}}^{*}$ that minimizes the matching error between the true object locations in the map in the set $M=\{\left(x_{m,i},y_{m,i}\right):i=1,2,\ldots ,p\}$ and the estimated location of the objects in set $M_{t}\left(\hat{q},Z_{t}\right)$:

(1) $$\begin{array}{ccc} {\hat{q}}^{*} & = & \left({\hat{x}}^{*},{\hat{y}}^{*},{\hat{\theta }}^{*}\right)\;=\;\arg\min_{\left(\hat{x},\hat{y},\hat{\theta }\right)}{\bar{h}}_{K}\left(M,M_{t}\left(\hat{q},Z_{t,k}\right)\right)\;=\;\frac{1}{K}\sum_{i=1}^{K}h_{i}\left(M,M_{t}\left(\hat{q},Z_{t}\right)\right)\; \end{array}$$

where ${\bar{h}}_{K}\left(A,B\right)$ is the modified Hausdorff distance computed with the *K* best-matching object coordinates between sets of coordinates A and B. For further details, please see [4].

Scan matching (1) based on the Iterative Closest Point (ICP) algorithm [32] as a metric for the distance between points in the map and the measurements set can also be employed instead of the modified Hausdorff distance to solve the localization problem.

## 4. Proposed Approach for Reliable Localization

A key aspect of the navigation in tunnels using the Hausdorff-based localization approach is the adequate definition of landmarks that ensure that the scan-matching problem can be solved unambiguously. To this end, the optimization of the landmarks’ geometry and positioning requires adequate parametrization. Two landmark models were considered and evaluated. Their characteristics are explained in the next subsections, together with the implementation aspects concerning the genetic algorithm search strategy, the simultaneous localization and mapping strategy, and the optimized landmark search process.

The optimization of the parameters that define a landmark’s geometrical characteristics for a given model is carried out using a genetic algorithm search strategy. The parameters of each landmark model, such as height, width, spacing of shape primitives, or the steps of a piecewise linear function (see Section 4.1 for specific details) are treated like genes that characterize an individual in a population of living organisms. The goal is to find a set of parameters (genes), which define the optimized genome or chromosome in the sense that the optimized genome is the one that delivers the best value of a fitness function (a performance or objective function of the optimization problem). In the context of robot localization, the fitness function can be defined as the total localization mean square error (MSE):

(2) $$\begin{array}{ccc} E & = & \frac{1}{N}\sum_{i=1}^{N}{\left(x_{i}^{g}-{\hat{x}}_{i}^{*}\right)}^{2}+{\left(y_{i}^{g}-{\hat{y}}_{i}^{*}\right)}^{2}+{\left(\theta_{i}^{g}-{\hat{\theta }}_{i}^{*}\right)}^{2} \end{array}$$

where $\left(x_{i}^{g},y_{i}^{g},\theta_{i}^{g}\right)$ are the ground truth values, and $\left(x_{i}^{*},y_{i}^{*},\theta_{i}^{*}\right)$ are the estimated pose values for samples i=1,2,…N of the robot’s trajectory along the tunnel with landmarks whose optimized geometry was found by the genetic algorithm search strategy. The ground truth values are available in simulation. In the validation experiments, the ground truth data are generated with an RTK-DGPS (real-time kinematic differential GPS) that delivers centimeter-level positioning accuracy (see Section 5 for specification details).

### 4.1. Landmark Parametrization

Two approaches for landmark generation are considered. The first one employs shape primitives as the basis for the definition of the genome. With this approach, the genetic algorithm seeks a combination of shape primitive that minimize the localization error in the solution of (1). The second approach defines the landmark as a piecewise linear function with points of varying heights. In this case, the genetic algorithm finds the height values that minimize the localization error. A difference with the approach based on shape primitives is that, in this second approach, the shape of the landmark is initially completely free and not conditioned by the selection of primitives.

Before introducing the landmark models, it is convenient to introduce the following definitions and notation. Let $H\left(x\right)\overset{def}{=}\left\{0\;\forall x<0;1\;\forall x\geq 0\right\}$ denote the Heaviside step function, the boxcar function of height *h* and width δ centered at *x* can then be defined as: ${⊓}_{h,\delta }\left(x\right)\overset{def}{=}h\cdot \left(H(x+\delta /2)-H(x-\delta /2)\right)$. Define the linear segment over interval (−δ/2,δ/2] starting at a height *a* and ending at height *b* as $l_{\delta }\left(x;a,b\right)=\left[\left(\left(b-a\right)/\delta \right)\left(x-\delta /2\right)+a\right]{⊓}_{1,\delta }\left(x\right)$.

#### 4.1.1. Landmarks Based on Shape Primitives

To model a landmark using shape primitives the following four functions were considered:

- Shape 1 (triangular): $s_{1}\left(x;W,H\right)\overset{def}{=}\wedge \left(x;W\right)=\left(1-|2x/W|\right){⊓}_{H,W}\left(x\right)$;
- Shape 2 (rectangular): $s_{2}\left(x;W,H\right)\overset{def}{=}{⊓}_{H,W}\left(x\right)$;
- Shape 3 (parabolic): $s_{3}\left(x;W,H\right)\overset{def}{=}\cap \left(x;W\right)=\left(1-{\left(2x/W\right)}^{2}\right){⊓}_{H,W}\left(x\right)$;
- Shape 4 (linear): $s_{4}\left(x;W,H\right)\overset{def}{=}l_{W}\left(x;0,1\right)H{⊓}_{H,W}\left(x\right)$

**Figure 2:**
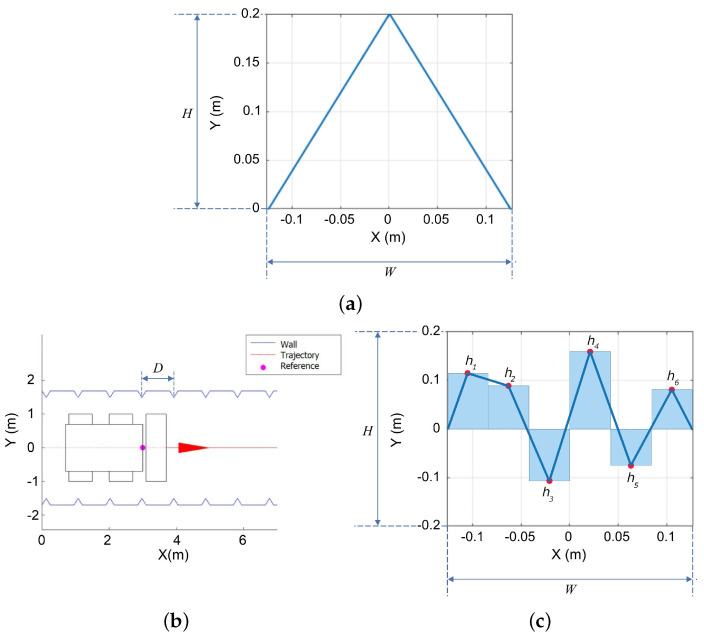
General size parameters of landmarks in terms of height *H*, width *W* in (**a**) and separation between landmarks *D* in (**b**). Shape parameters $h_{i}$, i=1,2,…,6 of the piecewise linear free shape landmarks define segment height (**c**).

The chromosome (sometimes called genotype or genome in the genetic algorithms literature) that represents an individual (a realization of a particular landmark) is defined in terms of genes that characterize the individual. A landmark model built using shape primitives has the following genome:

(3) $$\begin{array}{c} G_{P}=\left\{W,H,D\right\} \end{array}$$

where the genes correspond to the landmark’s width *W*, its height *H*, and the separation distance *D* between consecutive landmarks. The graphic representation of the width, height, and separation between landmarks is shown in Figure 2. Regardless of the shape geometry, whether it corresponds to a triangle, rectangle, parabola, or line, the shape can be bounded by a box of width *W* and height *H*, as shown in the case of the triangle in Figure 2a. Additionally, the separation between landmarks is defined by the reference distance *D*, which can be the same between all landmarks, as shown in Figure 2b or defined to randomly vary in a interval $\left[D^{*}-0.625,D^{*}+0.625\right]$ m, where $D^{*}$ is the optimized separation distance found with the search strategy explained in Section 4.4. In contrast, piecewise linear landmarks have six positions whose heights must be optimized together, with the overall width *W* and height *H* parameters, to produce an optimized shape with more degrees of freedom as illustrated in Figure 2c and explained in more detail in the next subsection.

#### 4.1.2. Landmarks Based on Piecewise Linear Functions

The *n*-segments landmark of width *W* can be defined as a piecewise linear function with points of height $h_{i}$, i=1,2,…,n as:

$$\begin{array}{ccc} L(x;W,H,h_{1},h_{2},\ldots ,h_{n}) & \overset{def}{=} & \sum_{k=1}^{n}H\;l_{\left(W/n\right)}\left(x-\left(k-1\right)W/n+W/2;h_{k-1},h_{k}\right) \end{array}$$

where $h_{0}=0$. It is to be noted that the landmark model $L(x;W,H,h_{1},h_{2},\ldots ,h_{n})$ is centered at x=0.

A landmark model built using a piecewise linear function has the following genome:

(4) $$\begin{array}{c} G_{L}=\left\{W,H,D,h_{1},h_{2},h_{3},h_{4},h_{5},h_{6}\right\} \end{array}$$

where the genes correspond to the landmark’s height points $h_{i}$, i=1,2,…,6, its width scaling *W*, its height scaling *H*, and the separation distance *D* between consecutive landmarks. A graphical representation of the piecewise linear landmark model is shown in Figure 2c. The range of values for each gene is summarized in Table 1 of the next section. The number of sections in the piecewise linear function considers the fact that polygonal shapes with more points for a given width *W* can be made smoother, but given sensor noise, additional smoothness does not provide additional distinctiveness. Using two or three segments would result in linear, triangular, or rectangular shapes already considered as part of the shape primitives. Therefore, to determine whether two consecutive shapes, e.g., two triangles or a triangle and a rectangle, offer an additional advantage, the piecewise linear free shape must have at least six parameters. Of course, it is possible to explore even more intricate geometries at the expense of an increased computational burden. Here, it was decided to limit the number of segments to six, but to compensate this limitation by also testing variants that can be easily computed, such as the horizontal symmetry and the vertically inverted landmark variations, as will be shown in the numerical computations and simulation Section 5.1.

### 4.2. Genetic Algorithm Implementation

Once *N* individuals characterized by chromosomes $G_{i}=\left\{g_{i,1},g_{i,2},\ldots ,g_{i,n}\right\}$, i=1,2,…,N of the form (3) or (4) have been initially created by sampling from a uniform distribution $U[g_{j}^{min},g_{j}^{max}]$ with lower and upper bound values $g_{j}^{min}$, $g_{j}^{max}$ from Table 1 for each gene $g_{i,j}$ to build an initial population, the genetic algorithm implemented iterates over the standard steps of fitness evaluation of each individual, selection of individuals, crossover (recombination) of individuals, mutation individuals, and insertion of offspring into the new generation as explained in [33,34]. The population size employed was of N=100 individuals. This number of individuals was empirically found to provide a good trade-off between ensuring a sufficiently large population for convergence while, at the same time, keeping computation time as low as possible.

The fitness evaluation function is the total localization MSE (2). The fitness score of each individual is employed to rank individuals, i.e., sort them in terms of ascending MSE. The selection of individuals employs a stochastic sampling known as stochastic universal sampling or systematic resampling [35,36], in which an initial random number $p_{0}\in U\left[0,1/N\right]$ is generated. Individuals laying along a line in which each one has a length proportional to its fitness value are selected by a pointer that takes constant size steps according to $p_{k}=\left(k-1\right)F/N+p_{0}$, where *F* is the total fitness (the sum of each individuals’ fitness) [35]. The reproduction step selects the best 5% of the total population and employs 75% of the remaining population for crossover. The crossover rule selects the genes (parameters) of consecutive parents according to a selection function in which a random binary vector of the length of the chromosome containing 0’s and 1’s is generated to indicate whether the gene value must be taken from one parent or the other. Next, the mutation step generates small random variations $\delta_{i,j}$ of the *i*-th child gene *j* by sampling a normal distribution $\delta_{i,j}∼N\left(0,\sigma^{2}\right)$, where $\sigma =(g_{j}^{max}-g_{j}^{min})/2$, and sets the gene $g_{i,j}$ of offspring *i* to a new value $g_{i,j}+\delta_{i,j}$. The reinsertion step simply creates a new population which includes the best 5% individuals and the remaining reproduced population. The number of offsprings generated in each iteration by crossover and mutation is such that the total amount of individuals *N* is kept constant from one iteration to the other. The stopping condition included a maximum number of iterations of 100, which was never reached because the condition on average relative decrease of the fitness function of 0.1% was met first, as shown in the results Section 5.

### 4.3. Simultaneous Localization and Mapping

For fast online computation, we employ an Extended Kalman filter and a likelihood field for map probability; see [37,38] for further details. The approach in [37], known as GMapping, is a popular algorithm that employs a Rao–Blackwellized particle filter to estimate the joint posterior. Our approach is similar to that of [38] in that it combines the scan matching and an adaptive update of the likelihood field instead of particle filters proposed in [37] to achieve similar results in terms of the root mean square (RMS) error and low execution time for practical real-time implementation.

**Figure 3:**
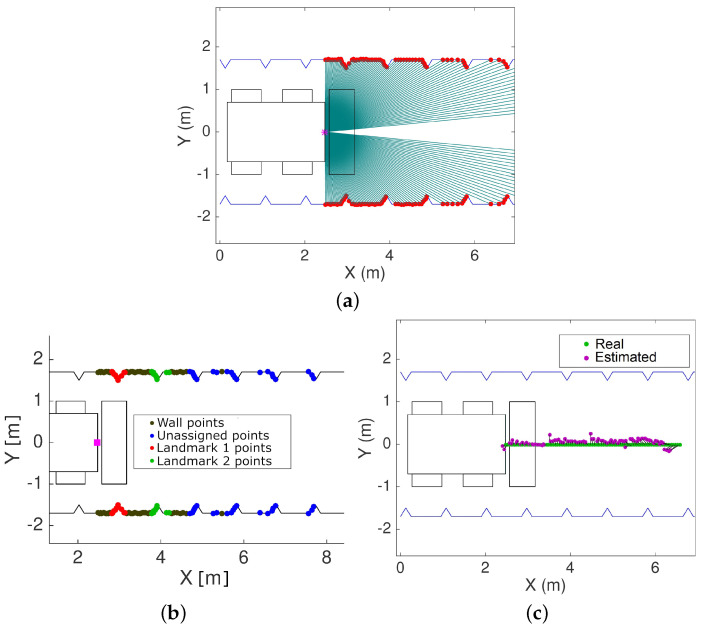
Top view of an ideal tunnel with triangular landmarks showing the matching of noisy lidar measurements to the map (**a**), the classification of landmark and wall points (**b**), and the estimated position using the proposed methodology (**c**).

In order to make the localization more efficient and accurate, the tunnel walls are removed in order to extract the landmarks and improve the localization’s accuracy. Tunnel walls may have some variability or roughness, but this variability is insufficient for unambiguous localization because the magnitude of the variability is comparable to the accuracy of commercially available lidars. Thus, tunnel walls are perceived as practically smooth straight or gradually curving walls. The background removal for landmark extraction is performed using the Random Sample and Consensus (RANSAC) algorithm [27,39]. To account for the possible curvature of the tunnel trajectory, tunnel walls are modeled as a cubic polynomial [40]. All points in the measurements set that do not fit the cubic polynomial within a tolerance margin are labeled as landmark points, as shown in Figure 3b, and are employed in the solution of the localization problem (1).

A top view of a machine moving along a tunnel is presented in Figure 3, which shows the matching of lidar measurements to the triangular landmarks in the map, and the resulting pose estimated using the Hausdorff-based scan-matching approach. To illustrate the matching process, Figure 3a presents an ideal predefined map consisting of lateral tunnel walls with symmetrically and equally spaced triangular landmarks. Despite these landmarks not being optimized in shape and separation, the simulated lidar measurements, including noise in range, are matched, minimizing the modified Hausdorff distance (1). Once the matching has been carried out, the landmarks are classified into wall and landmark measurements. The black points in Figure 3b correspond to wall points as detected by the RANSAC algorithm. The remaining points are treated as landmarks. The matching process considering only the landmarks yields the pose, i.e., position and orientation, thus solving the localization of the machine relative to the landmarks. The pose measurements obtained with the matching procedure can be filtered to generate position and orientation estimates, which are compared in Figure 3c.

### 4.4. Optimized Landmark Search Process

**Figure 4:**
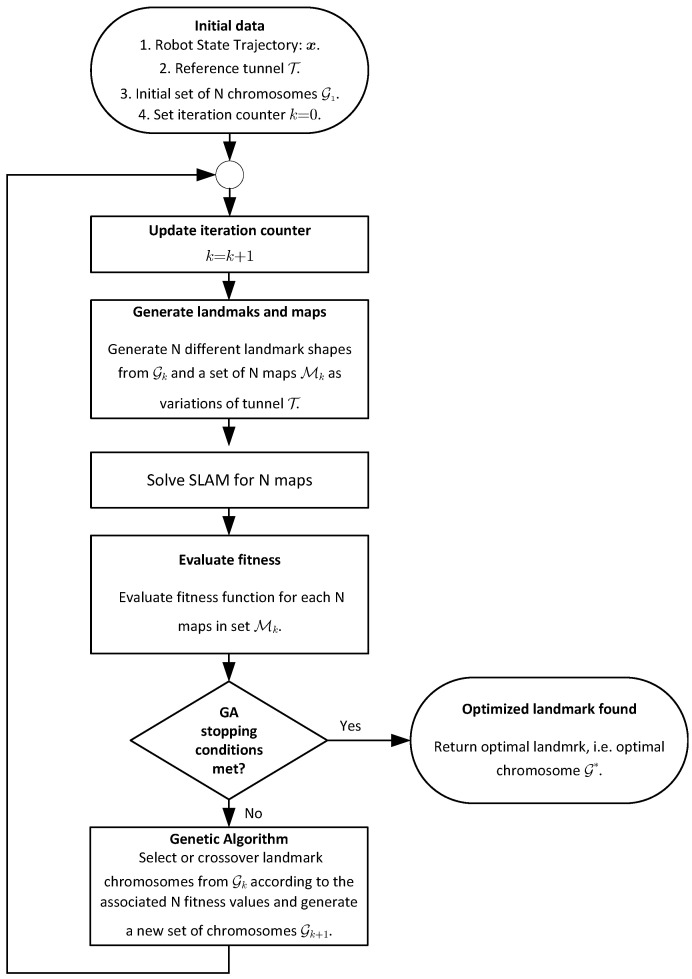
Implemented search scheme for optimized landmark geometries and spacing.

The process implemented to find the best landmark shapes and spacing is illustrated in Figure 4. The process starts by considering a reference tunnel without landmarks T, a known state trajectory of the robot moving along the tunnel x, and a set $G_{1}$ containing *N* individuals whose chromosomes or genomes define *N* tentative geometries and distances between landmarks. The initial set of chromosomes $G_{1}$ is employed to generate a first set $M_{1}$ containing *N* variations of tunnel T populated with landmarks according to the separation distance parameter. When creating the map, the spacing between consecutive landmarks $d_{\ell }$, ℓ=1,2,3,…, is drawn from a uniform distribution $d_{\ell }∼U\left[D^{*}-0.625,D^{*}+0.625\right]$ m, where the value $D^{*}$ is the value of the optimized landmark separation found in the previous iteration. Hence, the position of landmark ℓ=1,2,3,⋯, is defined as $p_{\ell }=p_{\ell -1}+d_{\ell }$ with $p_{0}=0$. The randomly varying distance in a bounded interval is important in order to avoid ambiguous matching of consecutive landmarks due to repeating landmark separations. Then, the SLAM problem is solved for the simulated robot following trajectory x in the *N* maps in $M_{1}$. The fitness function for the pose error (2) is evaluated for the *N* maps. Unless the stopping conditions explained in the subsection concerning the genetic algorithm implementation are met, the genetic algorithm must select the best candidates, produce crossover, and iterate until a chromosome $G^{*}$ defining the optimized landmark geometry is returned.

## 5. Results

The proposed approach is evaluated both in simulations and experimentally. The implementation of the robot trajectory simulation and SLAM, as well as the genetic algorithm, were implemented in Python without using other libraries than the standard mathematical function libraries NumPy and SciPy for numerical computations with arrays and matrices, integration of the ordinary differential equation of the robot’s dynamics using the odeint function. The motion model equations are explained in detail in [2] and describe motion dynamics of a semi-autonomous industrial compact skid-steer loader CAT 262C employed in the experiments. The simulations use a grid map with a 1 cm${}^{2}$ per pixel resolution and a position sensor model with a distance RMS error of 5 cm, which means 95% of the measurements are contained in an 8.65 m radius circle. The sampling frequency of the simulated system is 1 kHz and it is assumed that the same clock rate is employed for all sensors and the control loop. For the visualization of results, we use PyGame and Matplotlib libraries.

The experiments employ a semi-autonomous industrial compact skid-steer loader CAT 262C equipped with one Sensor STIM300 inertial measurement unit (IMU), two VectorNav IMU’s, one Piksi SwiftNav RTK-DGPS, two torque sensors by Manner Sensortelemetrie, four Sick LMS 511 lidars, wheel encoders, TE Connectivity MEAS inclination sensors, control, and navigation computer (running ROS Melodic, sampling sensors at 100 Hz) and wireless communication interfaces. The Sick LMS 511 lidar is designed for industrial operation outdoors even with dust or rain, allowing for multiple echoes and materials with different absorption/reflectance levels. The reflectance of soil/rocks is typically in the range of 50–60% [41,42], and given the laser beam power employed by LMS 511 and the manufacturer specifications [43], this lidar can scan soil or rocks up to 60–65 m without the aid of retro-reflective markers. In the experiments, we used common cardboard landmarks, which have a reflectance in the operating wavelength of lidar equivalent to that of soil/rocks [41].

The skid-steer loader in the test site is shown in Figure 1. The experiments were carried out in a mock-up of the tunnel without and with the optimized landmarks found in simulation to validate the approach. Following previously published work [44], we have selected the RMS error to assess the localization error.

**Figure 5:**
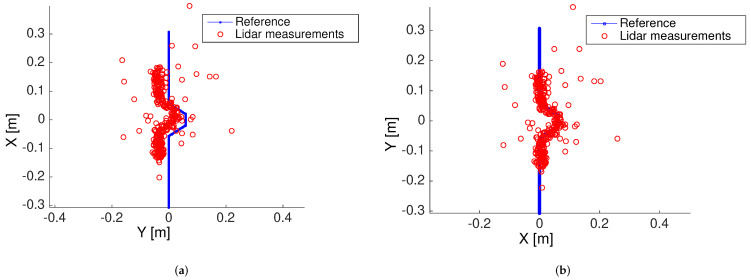
Scan-matching results using Hausdorff distance considering all measurements (**a**) and 80% of the best matching points (**b**).

The Hausdorff scan matching implemented in this work considered 80% of the best matching points that minimize the modified directed Hausdorff distance with respect to the reference model in order to improve the data association following the tuning recommendations in [4], i.e., *K* in (1) is set K=0.8m, where *m* is the total number of measurements. Since the scan matching procedure sorts the lidar measurements starting with the best fitting points, discarding the worst 20% of the matched points removes the matching bias and ensures sufficient measurements are available so that the matching does not become an ill-posed problem. An adaptive threshold *K* may be implemented in terms of an expectation-maximization strategy, but this aspect necessitates new theoretical developments beyond the scope of the current work to ensure the optimality of a dynamically adjusted threshold. To show that the choice of the fixed 80% threshold is adequate for practical applications, consider Figure 5, which presents the outcome of a simulation experiment in which 300 noisy lidar measurement points must be aligned to a reference model. The lidar ranging error is considered to have zero-mean Gaussian distribution with standard deviation σ=0.05 cm, which is a typical value for the Sick LMS 511 employed in our experimental validation. The Sick LMS 511 can deliver 720 scan points with an angular resolution of $0.25^{\circ }$ covering a 180${}^{\circ }$ field-of-view. Here, we are using less than half the points that may be obtained using Sick LMS 511 for testing purposes. In practice, the number of scan points covering a landmark will depend on the distance to the landmark and scanning angular resolution, which can be adjusted to different values between $0.042^{\circ }$ and $1^{\circ }$ in the case of Sick LMS 511. As shown in Figure 5a, when a 100% of the lidar measurements are employed, there exists a bias in the final alignment due to spurious measurements. On the other hand, when the 80% best-matching points are selected in the computation of the modified Hausdorff distance (1), the noisy point cloud is fitted more accurately to the reference model, as shown in Figure 5b.

### 5.1. Numerical Computation and Simulation Results

The robot simulation and the genetic algorithm to find the optimized landmarks were implemented in Python. The following trials were considered: P1—triangular primitive; P2—rectangular primitive; P3—parabolic primitive; P4—linear primitive; F1—piecewise linear free shape; F2—piecewise linear inverted free shape; F3—piecewise linear symmetric free shape; and F4—piecewise linear symmetric inverted free shape. If s(x) is a shape, then the inverted shape is 1−s(x). A symmetric shape is a shape that is an even function, i.e., s(x) is symmetric if s(x)=s(−x). In the implementation of the genetic algorithm, the parameters (genes of each individual’s chromosome) were allowed to take values in an interval whose lower and upper bounds are summarized in Table 1.

**Figure 6:**
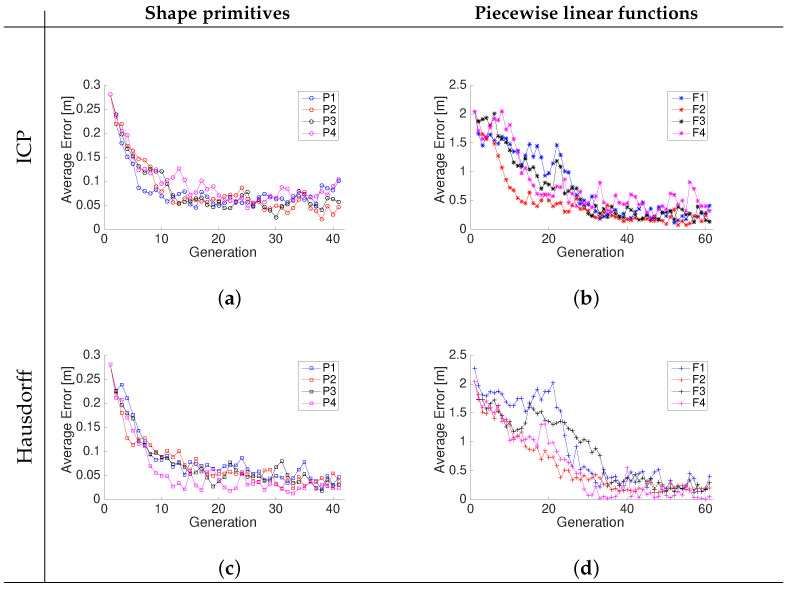
Convergence of the square root of the trace of the matching error covariance matrix for shape primitives (**a**,**c**) and piecewise linear functions (**b**,**d**) using ICP and Hausdorff matching, respectively.

**Figure 7:**
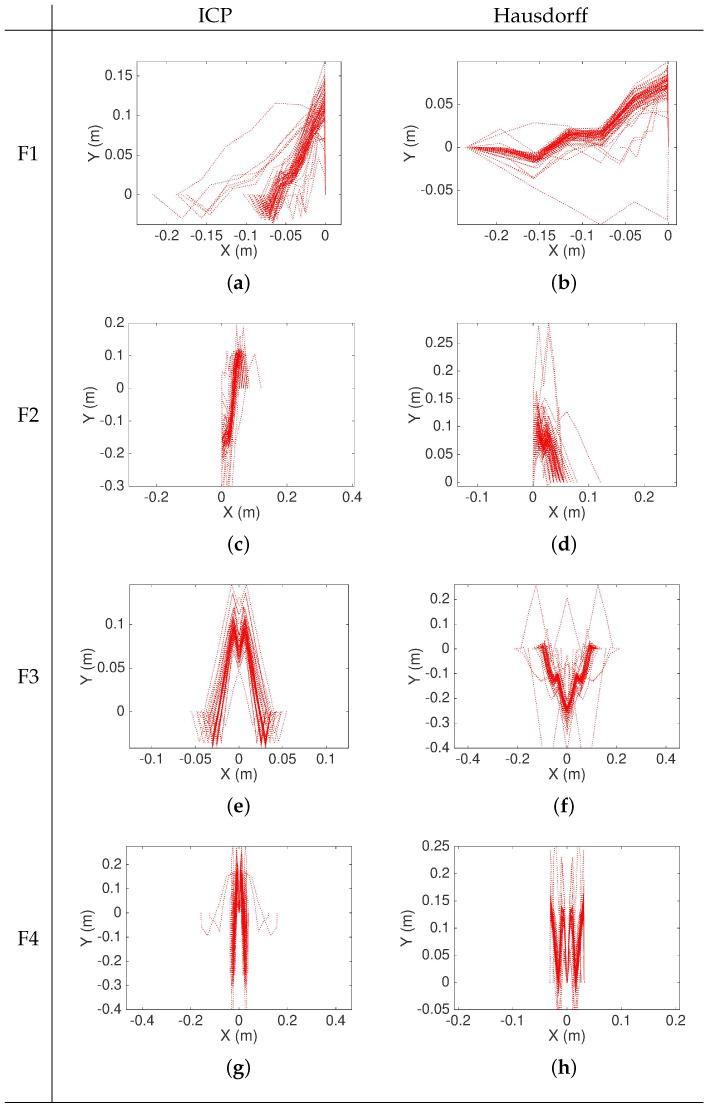
Optimized piecewise linear shapes found by the genetic algorithm after 45 iterations: F1—free (**a**,**b**); F2—inverted (**c**,**d**); F3—symmetric free shape (**e**,**f**); F4—symmetric inverted (**g**,**h**). The graphs show the evolution of multiple iterations superimposed showing the convergence to the optimized landmark geometry.

The convergence of the RMS position error component of fitness function for each iteration of the genetic algorithm while searching for an optimized landmark geometry and separation is shown in Figure 6. The resulting piecewise linear models are shown in Figure 7. The different curves that are shown in each graph of Figure 7 represent a realization of the best individual’s chromosome for a given generation. After several iterations, the best individuals of each generation evolve and converge to overlapping shapes that strongly coincide, thus confirming that an optimized geometry minimizing the pose error (2) exists. It is to be noted that the relative average decrease in the fitness function (2) becomes less than 0.1% for either the ICP or Hausdorff matching approaches after 20 iterations when using the shape primitives and at least 40 iterations when using the linear piecewise landmark model because it has more parameters. The optimized landmarks found in iteration 45 for the different shapes and models were selected when testing the localization performance to make a fair comparison and remove the differing amount of iterations as a possible advantage factor.

**Figure 8:**
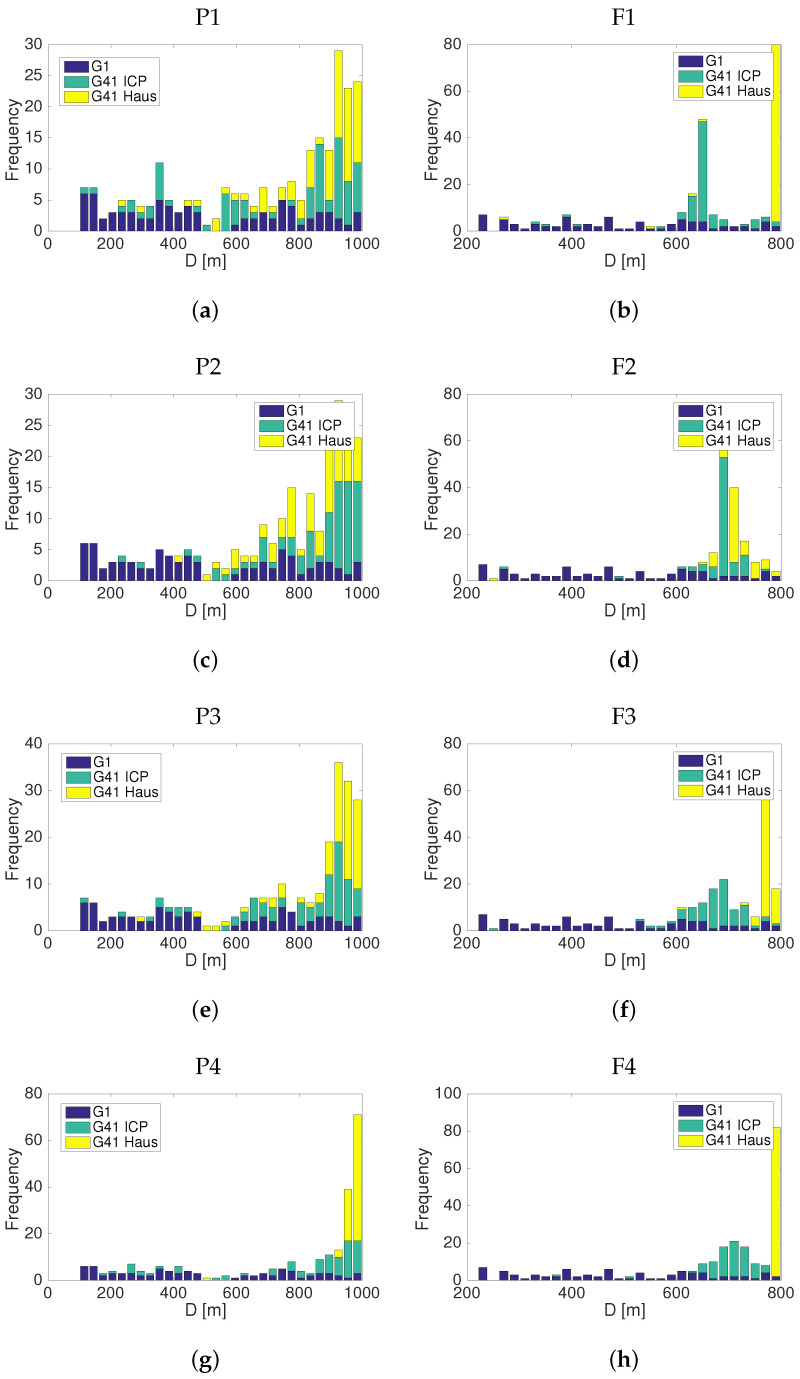
Histogram of the optimized distances between landmarks found by the genetic algorithm after 41 iterations (G41) compared to the initial distribution of distance genes (G1) for the primitive shapes P1, P2, P3, P4 (**a**,**c**,**e**,**g**), and the free piecewise linear shapes F1, F2, F3, F4 (**b**,**d**,**f**,**h**).

Regardless of the type of landmark, the results Figure 6 show that the Hausdorff matching converges with less variability than ICP. The genetic algorithm not only identified the best shapes for accurate matching, but also identified the optimal distance *D* between the landmarks, which was found to be between 8 and 10 m. Figure 8 shows that for the different landmarks, the initial proposed distance values *D* are approximately uniformly distributed. Regardless of the matching approach (ICP or Hausdorff), the distribution after 41 iterations of the *gene* associated to the separation *D* between landmarks concentrates around 9–10 m when using the shape primitives P1, P2, P3, or P4 landmarks, and they around 7–8 m when using the piecewise linear free shape landmarks F1, F2, F3, and F4. It is to be noted that in the case of landmarks F1, F2, F3, and F4, ICP tends to prefer closer landmarks with D≈7 m, while the Hausdorff matching produces lower RMS localization errors with landmarks separated by D≈8 m, as shown in Figure 8.

From the simulations presented in Table 2, it is possible to confirm that the triangular shape model (P1) yields the smallest RMS localization error for the robot in a simulated tunnel that was 10 m long, with an error of 22 mm using the Hausdorff matching strategy. The second best landmark is the symmetric inverted piecewise linear model (F4) resembling an inverted double triangular shape or “W” shown in Figure 7h, which yields an RMS localization error of 24 mm using the Hausdorff matching strategy. The results in Table 2 show that the best results are achieved with the Hausdorff matching strategy when compared to the ICP method. Even if ICP had a better performance than the Hausdorff matching strategy with two of the linear piecewise models, the Hausdorff matching technique delivers better results in all other cases because the RMS errors are 30–70% smaller.

### 5.2. Experimental Validation

The experimental validation using the semi-autonomous CAT 262C skid-steer loader consisted of 15 repetitions each, first in a 10 m mockup tunnel without landmarks (experiment 1), then using the triangular shape primitive model P1 identified by the genetic algorithm (experiment 2), and, finally, the symmetric inverted piecewise linear landmark model F4 (experiment 3). The localization was solved with both the ICP and the Hausdorff matching strategy. The results in terms of average RMS localization error and 95% confidence intervals are summarized in Table 3. The experimental results reported in Table 3 employed the best landmarks evaluated in simulation as reported in Table 2, which are landmarks P1 (triangular shape primitive) and F4 (symmetric inverted piecewise linear free shape).

The experimental results confirm that the symmetric inverted landmark F4 is slightly better compared to the triangular shape landmark model P1. However, the 26 mm difference on average is within the 95% confidence interval, which for the symmetric inverted landmark, is 93 mm. Compared to the case with no landmarks, which has an RMS localization error of almost twice the traveled distance (10 m) in the experiments, the localization approach with the proposed landmarks is very accurate and proves to be suitable for the localization of underground mining loaders and trucks. It is also to be noted that ICP performed better with an RMS localization error 13 mm smaller than the RMS localization error obtained with the Hausdorff matching strategy using the symmetric inverted landmark model F4. However, with the simpler triangular landmark model P1, ICP yields an RMS localization error that is 23 mm larger. Comparing the RMS errors presented in Table 2 and Table 3, it is possible to observe that the experimental RMS positioning error is approximately 10 times larger than the RMS positioning error obtained in the simulations. This is mainly explained by the fact that the performance of the RTK-DGPS had, in practice, an RMS error of 8.3 cm, which means that about 95% of the measurements fall within a circle with a 14.4 cm radius. On the other hand, the clock rate of the different subsystems is different. The control loop was implemented at 100 Hz, but the RTK-DGPS provides measurements at 10 Hz, while the lidar and RTK-DGPS have 10 Hz sampling rates. Since the RTK-DGPS measurements are employed as ground truth, the practical RMS error includes the GPS error, but also the lidar’s accuracy, which are approximately 5 cm.

### 5.3. Validation with an Underground Mine Dataset

**Figure 9:**
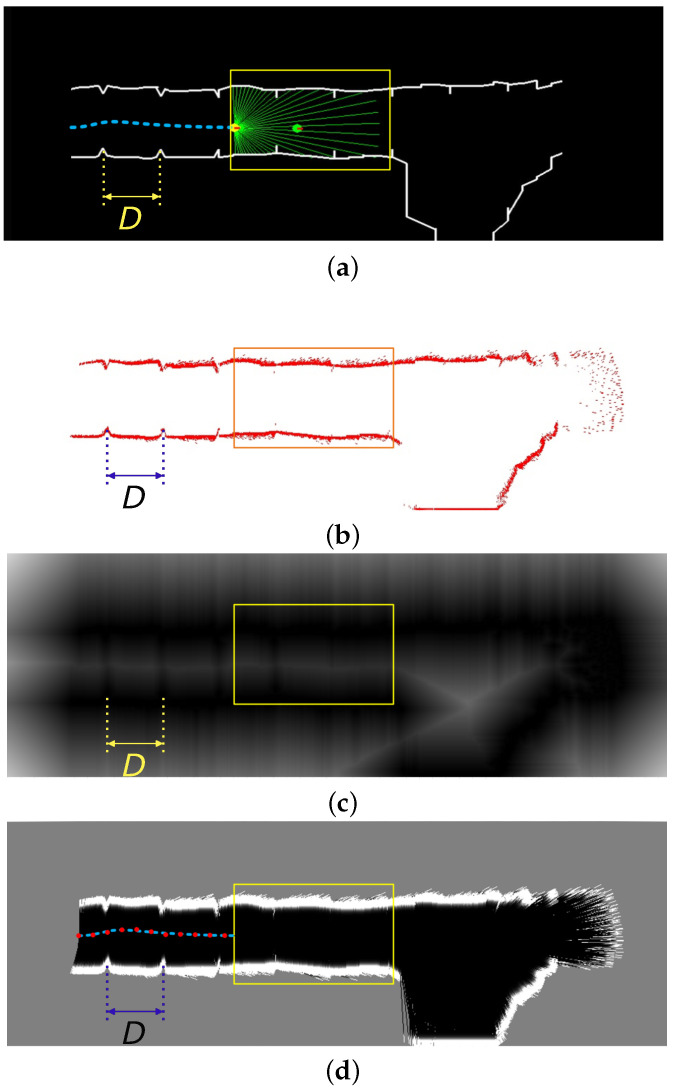
SLAM solution of the El Teniente tunnel showing the ground truth map, trajectory, and distance between landmarks (**a**); the laser rangefinder scan (**b**); the Voronoi distance transform of the scan (**c**); and the estimated grid map and trajectory results (**d**).

A validation of the approach and the optimized landmarks is also carried out using the publicly available 3D point cloud dataset of the El Teniente copper mine located in Chile [8]. A 100 m section of one of the tunnels was extracted from the dataset and artificial landmarks P1 were added with randomly varying distances D∼U[9.25,10.5] m around the optimized value found by the genetic algorithm to ensure non-uniform spacing between landmarks and thus avoid ambiguous matching of consecutive landmarks due to repeating landmark separations. The triangular landmark geometry P1 was chosen for validation with the data underground mine data set because it is a simpler geometry to manufacture and because it yielded an RMS localization error in the real-world experiments that is similar to that of the best landmark geometry F4 (see Table 3). Furthermore, the RMS localization error obtained in the runs of the Genetic Algorithm give a slight advantage to P1 over F4, when using the Hausdorff matching strategy, as shown in Table 2. A physically accurate model of the skid-steer loader developed in [2] was simulated to evaluate the effectiveness of the landmarks for SLAM using the scan matching procedure based on the modified Hausdorff distance [4]. The results are shown in Figure 9, which shows the traversed trajectory in Figure 9a, the matched point clouds in Figure 9b, the distance transform of the point clouds employed for matching using the modified Hausdorff distance in Figure 9c, and the resulting map and measured trajectory (red) compared to the trajectory ground truth (blue) in Figure 9d. The ground truth corresponds to the skid-steer loader’s trajectory obtained by the model simulation assuming noise-free position sensors. On the other hand, the map considers a grid with a resolution of 10 × 10 cm${}^{2}$ per pixel, while the measurement model considers the ranging error to be zero-mean Gaussian distributed with standard deviation σ=0.05 cm, which is a typical value for the Sick LMS 511. An RMS localization error between the true position and the measured position of 0.163 ± 0.072 m was obtained after 15 repetitions, i.e., the simulation was repeated 15 times with a virtual machine driving in the tunnel considering the sensor noise parameters. The obtained localization error was registered to compute the RMS error across the 15 realizations and the 95% confidence interval of the RMS localization error. It is to be noted that without the landmarks, it is not possible to solve the SLAM problem correctly because the tunnel walls are almost smooth, thus causing the matching to diverge due to the lack of anchor points that could be used for reliable scan alignment. The RMS localization error in the dataset without landmarks obtained with the Hausdorff matching approach was 194.3 ± 0.22 m, while IPC resulted in an RMS localization error of 201.7 ± 0.15 m.

## 6. Discussion

The main findings after the experimental validation of the proposed strategy for navigation in mining tunnels are discussed as follows, considering both their significance and limitations:

1. The approach based on optimized artificial landmarks’ geometry and spacing is suitable for localization and mapping in smoothly bored underground mining tunnels, where no GPS signal is available and where deploying and maintaining a network of active RF or optical beacons is costly and difficult.
2. Without landmarks, it is not possible to solve the localization problem using lidar information in smooth tunnels because the localization problem becomes ill-posed, as evidenced by the cumulative error of the positioning without landmarks reported in Table 3. Even if different SLAM techniques have been developed to reduce the well-known localization slip or drift problem [45], reliable underground localization and mapping requires accurate positioning drift-free strategies [7] to ensure industrial grade safety standards. Therefore, artificial landmarks are an essential part of the proposed solution for operation in adverse and challenging underground mining conditions. Other solutions, relying on SLAM algorithms and variants that employ natural landmarks may work partially and exhibit drift sporadically; thus, the use of natural landmarks is still not applicable for 24/7 working schedules required by the mining industry. On the other hand, passive artificial landmarks may be cheaper to manufacture, install, and maintain compared to active RFID or IR beacons.
3. The optimization of landmark geometries for the different models (shape primitives and piecewise linear) yields expected positioning errors in the range 20–90 mm depending on the geometry. Considering the approximately 50 mm difference between the worst and best model, it is possible to conclude that adequate landmark design and optimization is worth the effort.
4. In addition to the development of an optimization scheme for the landmarks’ geometry and spacing presented in Section 4.4) to improve localization, important contributions that are of practical relevance are the validation of: (i) the feasibility of the approach through experimental validation for localization in relatively smooth tunnels, in which traditional scan matching and visual features do not work due to the lack of sufficiently distinctive features that could be matched without ambiguity (see Table 3); (ii) the advantage of Hausdorff-based matching compared to the ICP method (see Table 2); and (iii) the gains in localization accuracy than can be achieved by optimizing the geometry and spacing of landmarks by means of a genetic algorithm search strategy (see Table 2).
5. The experiments were conducted with a mock-up of a smooth tunnel both with and without landmarks. Modern machine-bored tunnels are relatively smooth and lack features. Thus, the mock-up replicates a challenging geometry for matching and localization rather than visual appearance. In order to further validate the approach, an accurate 3D model of a 100 m section of one of the tunnels of the Chilean El Teniente copper mine from the dataset by [8] was employed. Fifteen iterations assuming typical motion disturbances and sensor noise, with magnitudes equivalent to those of the CAT 262C [2] and Sick LMS 511 lidar, were carried out to ensure statistical significance. Future work considers creating a new dataset and additional testing in different underground tunnels, which during this research has not been possible due to increased restrictions to access mining sites during the pandemic.
6. The RMS positioning error obtained in the experimental validation of Section 5.2 is influenced by the accuracy of the RTK-DGPS (RMS error of approximately 8.3 cm), which was employed as the ground truth. Another limitation arises from the accuracy and resolution of the lidar scanner, which is approximately 5 cm. We expect that the positioning accuracy measured in our experiments should improve with ongoing technological advances and the development of more accurate lidar and GPS sensors.
7. Concerning the practical implementation of the approach, two important aspects need to be considered: (i) the execution time and (ii) the environment’s visibility conditions. The results presented in Section 5.3 show that the execution time is adequate for real-time implementation applicable to underground machines operating at standard speeds of 20 to 30 km/h. The effects of environmental visibility due to dust were not tested as part of this study. However, there exist laser range scanners and other vision systems that have been successfully employed in commercial collision avoidance systems for mining equipment, e.g., SICK’s MINESIC100 EPS, MINESIC100 TCW or Visionary-B.
8. The accurate localization of artificial landmarks on the map does not need to be performed using accurate georeferencing or topographic stations since the landmark’s location can be jointly estimated with the position. Once the landmarks have been deployed, practically no maintenance is required unless some are damaged and need to be replaced. The low-maintenance requirements are an advantage of the proposed solution compared to systems requiring energy supply and network connectivity for active optical and RF beacons.
9. Our ongoing research efforts are focused on improving the proposed approach with deep learning techniques and neural networks for different purposes, which include visual feature extraction, scene recognition, ego-motion estimation, and map matching. Techniques based on deep neural networks have shown promising results to improve lidar matching, e.g., OverlapNet by Chen et al. (2021) [46], and optical flow estimation, e.g., Flownet by Fischer et al. (2015) [47], including RGB-D SLAM with convolutional neural networks [48] and 3D indoor scene mapping [49]. Hence, these techniques may improve the accuracy and robustness of lidar and visual matching, as well as motion estimation, which are essential for SLAM in underground tunnels. It is to be noted that an important challenge for the application of visual techniques in the harsh mining environments is the poor visibility in tunnels due to low illumination conditions and dust, as well as machine vibrations, which are typically not a problem in indoor or urban robotics.

## 7. Conclusions

An approach for reliable autonomous navigation in modern smoothly bored mining tunnels which ensures drift-free localization and consistent mapping has been developed and validated. The approach relies on the optimization landmark geometry and positioning (distance between landmarks). Finding the optimized parameters was achieved with a genetic algorithm search strategy. The results show that optimizing a free shape using a piecewise linear function leads to a inverted double triangular shaped landmark, while very similar results are obtained with the optimized triangular shape primitive. From a practical perspective, it may be more convenient to use simple optimized triangular-shaped landmarks because the positioning accuracy is on average around 22 cm, with a small difference of 2.6 cm, which is within the ±9.3 cm confidence interval of the piecewise linear inverted double triangular shape. The experimental validation using a compact skid-steer excavator CAT 262C shows that without landmarks, the cumulative drift error steadily grows, and correct localization is not possible due to the ambiguity in lidar scan matchings. The experimental results thus confirm that using shape-optimized passive landmarks are a reliable alternative for localization and navigation in modern underground smoothly bored mining tunnels, for which electrically powered active optical or RF beacons are less likely to be accepted by the underground mining industry due to concerns on maintenance cost involved to prevent malfunctioning risks and ensure operational safety in case of a loss of power supply. The applicability of the localization approach for SLAM in real underground mines was verified using an accurate 3D model of a 100 m tunnel section of El Teniente mine in Chile, which is the largest underground copper mine in the world. Ongoing research is concerned with improving the accuracy and robustness of the proposed localization and mapping approach with deep learning techniques for ego-motion estimation, map matching, and the extraction of visual features that could be used as landmarks. An important challenge for the application of visual techniques in the harsh mining environments is the poor visibility in tunnels due to low illumination conditions and dust, as well as machine vibrations, which are typically not a problem in indoor or urban robotics. Our work in progress also considers improvements to the proposed approach for navigation in fruit groves and forests with large tree canopies that create tunnel-like conditions.

## Figures and Tables

**Table 1:** Minimum and maximum parameter values for the landmark models.

			Parameter Values (Genes)								
Landmark Model		Units	*H*	*W*	*D*	$h_{1}$	$h_{2}$	$h_{3}$	$h_{4}$	$h_{5}$	$h_{6}$
Primitive shape	Min.	m	0.01	0.01	0	-	-	-	-	-	-
	Max.	m	0.30	0.60	100	-	-	-	-	-	-
Piecewise linear	Min.	m	0.01	0.05	0	−2.00	−2.00	−2.00	−2.00	−2.00	−2.00
	Max.	m	0.35	0.150	750	2.00	2.00	2.00	2.00	2.00	2.00

**Table 2:** RMS localization error for each type of landmark found by the genetic algorithm.

	RMS Error [m]			RMS Error [m]	
Primitive Shape Models	ICP	Hausdorff	Piecewise Linear Model	ICP	Hausdorff
P1	0.042	0.022	F1	0.073	0.052
P2	0.091	0.026	F2	0.043	0.064
P3	0.063	0.028	F3	0.033	0.068
P4	0.065	0.026	F4	0.035	0.024

**Table 3:** RMS localization error using the optimized landmarks during experimental validation.

		RMS Localization Error [m]	
Experiment		ICP	Hausdorff
1	Without landmarks	20.765 ± 0.074	19.748 ± 0.113
2	P1—Triangular shape landmark	0.258 ± 0.046	0.235 ± 0.035
3	F4—Symmetric inverted landmark	0.206 ± 0.096	0.219 ± 0.093

## Data Availability

Mining tunnel 3D lidar data for "El Teniente" mine used in this paper are publicly available at [8].

## Abbreviations

The following abbreviations are used in this paper:

AGV	Automated Guided Vehicle
AMR	Autonomous Mobile Robot
DGPS	Differential GPS
EKF	Extended Kalman Filter
EM	Expectation-maximization algorithm
F1	Piecewise linear free shape
F2	Piecewise linear inverted free shape
F3	Piecewise linear symmetric free shape
F4	Piecewise linear symmetric inverted free shape
GCS	Guidance control system
GPS	Global Positioning System
ICP	Iterative Closest Point
IMU	Inertial measurement unit
LC	Inductive-capacitive
LGV	Laser-guided vehicle
LHD	Load-haul and dump
MSE	Mean square error
NDT	Normal Distribution Transform
P1	Triangular primitive
P2	Rectangular primitive
P3	Parabolic primitive
P4	Linear primitive
RANSAC	Random Sample and Consensus
RMS	Root mean square
RTK	Real time kinematic
SLAM	Simultaneous localization and mapping
